# Enhanced Expression of microRNA-1273g-3p Contributes to Alzheimer’s Disease Pathogenesis by Regulating the Expression of Mitochondrial Genes

**DOI:** 10.3390/cells10102697

**Published:** 2021-10-09

**Authors:** So Hee Kim, Kyu Yeong Choi, Yega Park, Catriona McLean, Jiyu Park, Jung Hoon Lee, Kyung-Hwa Lee, Byeong C. Kim, Yun Hyun Huh, Kun Ho Lee, Woo Keun Song

**Affiliations:** 1 Cell Logistics Research Center, School of Life Sciences, Gwangju Institute of Science and Technology, Gwangju 61005, Korea; sohee@gist.ac.kr (S.H.K.); dprk94@gmail.com (Y.P.); yenjiyu@gm.gist.ac.kr (J.P.); 2 Gwangju Alzheimer’s Disease and Related Dementia Cohort Research Center, Chosun University, Gwangju 61452, Korea; khaser@gmail.com; 3 Department of Pathology, The Alfred Hospital, Melbourne, VIC 3004, Australia; C.McLean@alfred.org.au; 4 Department of Biochemistry and Cell Biology, Geisel School of Medicine, Dartmouth College, Hanover, NH 03755, USA; Junghoon.Lee.Gr@dartmouth.edu; 5 Department of Pathology, Chonnam National University Research Institute of Medical Science, BioMedical Sciences Graduate Program, Chonnam National University Hwasun Hospital and Medical School, Gwangju 58128, Korea; mdkaylee@jnu.ac.kr; 6 Department of Neurology, Chonnam National University Medical School, Gwangju 61469, Korea; byeong.kim7@gmail.com; 7 School of Life Sciences, Gwangju Institute of Science and Technology, Gwangju 61005, Korea; yhuh@gist.ac.kr; 8 Department of Biomedical Science, Chosun University, Gwangju 61452, Korea; 9 Aging Neuroscience Research Group, Korea Brain Research Institute, Daegu 41062, Korea

**Keywords:** Alzheimer’s disease, amyloid β, miR-1273g-3p, plasma, cerebrospinal fluid, mitochondria, oxidative stress, TIMM13

## Abstract

Alzheimer’s disease (AD) is the most common form of dementia in the elderly population, but its underlying cause has not been fully elucidated. Recent studies have shown that microRNAs (miRNAs) play important roles in regulating the expression levels of genes associated with AD development. In this study, we analyzed miRNAs in plasma and cerebrospinal fluid (CSF) from AD patients and cognitively normal (including amyloid positive) individuals. miR-1273g-3p was identified as an AD-associated miRNA and found to be elevated in the CSF of early-stage AD patients. The overexpression of miR-1273g-3p enhanced amyloid beta (Aβ) production by inducing oxidative stress and mitochondrial impairments in AD model cell lines. A biotin-streptavidin pull-down assay demonstrated that miR-1273g-3p primarily interacts with mitochondrial genes, and that their expression is downregulated by miR-1273g-3p. In particular, the miR-1273g-3p-target gene TIMM13 showed reduced expression in brain tissues from human AD patients. These results suggest that miR-1273g-3p expression in an early stage of AD notably contributes to Aβ production and mitochondrial impairments. Thus, miR-1273g-3p might be a biomarker for early diagnosis of AD and a potential therapeutic target to prevent AD progression.

## 1. Introduction

Alzheimer’s disease (AD) is the most common type of dementia. It is pathologically characterized by the accumulation of extracellular amyloid β (Aβ) plaques and intracellular neurofibrillary tangles in the brain. AD patients show memory loss and cognitive and behavioral impairments caused by massive neurodegeneration mainly at the medial temporal lobe, which contains the hippocampus and surrounding cortical regions [1,2]. Aβ peptides produced by the sequential proteolysis of amyloid precursor protein (APP) by γ-secretase and β-site APP cleaving enzyme 1 (BACE1) have long been thought to be a key factor that initiates neurodegeneration in AD prior to NFT formation [3,4]. However, the mechanisms underlying Aβ production and deposition in sporadic AD patients are still poorly understood. 

Recently, mitochondrial dysfunction has gained attention in the context of Aβ production and neurodegeneration [5,6]. When mitochondrial activity is defective, insufficient energy production and reactive oxygen species overload induce cellular stress and eventual cell death [7]. Studies showed that the expression of mitochondrial enzymes and the oxygen consumption rate (OCR) were significantly decreased and that oxidative stress molecules, such as 8-hydroxyguanosine and nitrotyrosine, were increased in early-stage AD patients [8,9]. In the 3xTG-AD mouse model, mitochondrial respiration was markedly reduced prior to the appearances of cognitive impairment, Aβ plaques, or NFTs [10]. Several other studies demonstrated that impairment of mitochondrial function could induce Aβ production and neurodegeneration. In particular, cybrid cells containing mitochondria obtained from AD patients showed decreased mitochondrial activity and increased production of Aβ [11,12,13]. In addition, mitochondrial dysfunction by inhibitors of oxidative phosphorylation complexes facilitated Aβ production [14,15], and oxidative stress induced the expression of BACE1 [16,17,18]. Accordingly, studies focusing on the mechanism(s) underlying mitochondrial dysfunction in AD will surely help us unravel the cause of AD pathogenesis.

MicroRNAs (miRNAs), small non-coding RNAs, are known as an important regulator of gene expression in various physiological processes [19]. Many miRNAs are associated with Aβ processing or NFT formation, and their expression levels are shown to be differentially regulated in AD [20,21]. miRNAs stably exist in body fluids, either in their free form or packaged into vesicles [22,23,24]. Monitoring miRNAs in cerebrospinal fluid (CSF), which is in direct contact with brain tissue, is considered to be a promising strategy for detecting early events of brain diseases [25,26]. Studies on the levels of miRNAs in blood or CSF from early-stage AD patients revealed that the plasma levels of miR-92a-3p, miR-181c-5p, and miR-210-3p were upregulated in mild cognitive impairment (MCI) and AD patients [27]. Another study analyzed changes in the level of miRNAs in CSF according to various Braak stages or plaque density stages to determine AD-associated miRNAs [28]. A meta-analysis of miRNAs in brain, blood, and CSF identified miRNAs that appeared to be consistently associated with AD progression [29]. However, we know relatively little about miRNAs in early-stage AD patients and how the altered miRNAs might function in AD progression. 

In this study, we report that miR-1273g-3p is significantly upregulated in CSF of early-stage AD patients, as assessed by microarray and qPCR analyses. A pull-down experiment performed using biotinylated-miR-1273g-3p revealed that miR-1273g-3p primarily interacts with mitochondrial genes and downregulates their expression levels. Here, miR-1273g-3p causes mitochondrial dysfunction and subsequent aspects of AD pathogenesis, such as neurodegeneration and the formation of Aβ plaques.

## 2. Materials and Methods

### 2.1. Human Plasma and CSF Samples

Human plasma and CSF samples from 195 participants aged 65–90 years were obtained from the National Research Center for Dementia at Chosun University in Gwangju, Korea. All participants were tested for a full dementia screening, including medical history, neurological examination, laboratory and neuropsychological tests, and brain MRI. The clinical diagnosis was made according to the criteria of the National Institute of Neurological and Communicative Disorders and Stroke–Alzheimer Disease and Research Disorders Association [30]. Participants in cohort 1 for microarray of plasma miRNAs consisted of amnestic MCI (aMCI), AD patients and cognitively normal controls. Participants for qPCR analysis of miRNAs in plasma (cohort 2) and CSF (cohort 3) additionally underwent brain amyloid-PET imaging using ^18^F-florbetaben and CSF analysis of Aβ40/42 and pTau^181^/Tau within 1 year from the screening test. The clinical and demographic information of participants for this study is presented in Table 1.

Plasma samples were prepared twice by centrifugation (3000 rpm, 4 °C, 10 min) of fasting blood samples in EDTA vacutainer within 20 min after collection and immediately stored at −80 °C until use. CSF samples were collected in Falcon polypropylene tubes (BD Biosciences, Franklin Lakes, NJ, USA), centrifuged for 10 min at 2000 rpm at 4 °C and immediately stored at −196 °C liquid nitrogen until use.

### 2.2. Human Postmortem Brain Samples

Formalin-fixed paraffin embedded brain samples were obtained from the Victorian Brain Bank Network (Victoria, Australia) and Chonnam National University Hwasun Hospital and Medical School (Jeollanam-do, Korea). The samples used in our experiment were derived from 8 AD patients (64–88 years, 6 males and 2 females) and 5 cognitively normal controls (64–79 years, 4 males and 1 female).

### 2.3. Cell Culture

H4 cell line expressing APPswe^mut^ (H4-APPswe) were gifted from Dr. Jung-Hyuck Ahan in Ewha Womans University School of Medicine, Seoul, Korea. H4-APPswe cells were maintained in Dulbecco modified Eagle’s medium (DMEM, Gibco, Amarillo, TX, USA) with 10% fatal bovine serum (Hyclone, Logan, UT, USA), 1X Antibiotic-Antimycotic (Gibco) and 500 ug/mL geneticin (Gibco). SH-SY5Y cell line (ATCC) were maintained in DMEM supplemented with 10% FBS, 1x Antibiotic-Antimycotic. All cell lines were cultured in a humidified incubator containing 95% air/5% CO_2_ at 37 °C and routinely tested for mycoplasma using MycoAlert mycoplasma detection kit (Lonza, Basel, Switzerland). For transfection, about 25,000/cm^2^ H4-APPswe cells or 40,000/cm^2^ SH-SY5Y cells were plated on 6-well plate or cover glasses in 12-well plate, and transfected next day. For transfection, about 25,000/cm^2^ H4-APPswe cells or 40,000/cm^2^ SH-SY5Y cells were plated on 6-well plate or cover glasses in 12-well plate, and transfected next day.

### 2.4. Plasmids, miRNA Mimic and Inhibitor and Transfection

An amount of 75 nM (unless otherwise stated) miR-1273g-3p mimic and mimic negative control (Dharmacon, Lafayette, CO, USA); 10 nM miRCURY LNA miR-1273g-3p power inhibitor and inhibitor control (Qiagen, Hilden, Germany); and 50 nM ON-TARGETplus siRNAs targeting TIMM13 and non-targeting control siRNA (Dharmacon) were transfected into cells using DharmaFECT 1 reagent (Dharmacon). The 3′UTR of GLRX5, MTCH1, VDAC2 and TIMM13 and coding sequence of TIMM13 were amplified by PCR using cDNA of SH-SY5Y cells as template and inserted into pEGFP C1 and pcDNA 3.0 vector, respectively, using EZ-cloning kit (Enzynomics, Daejeon, Korea). The primers are described in Table S4. Plasmids were transfected into cells using Lipofectamine 3000 (Invitrogen, Waltham, MA, USA).

### 2.5. Microarray

Each 1ml of plasma from 4 participants was used for microarray. Total RNA was isolated using miRNeasy serum/plasma kit (Qiagen) following the manufacturer’s instructions and concentrated by ethanol precipitation method. After a quality check using Bioanalyzer 2100 (Agilent, Santa Clara, CA, USA), 1 μg total RNA was labeled using the FlashTag^TM^ Biotin HSR RNA Labeling kit (Affymetrix, Santa Clara, CA, USA), hybridized to Affymetrix GeneChip miRNA array 4.0. and scanned with an Affymetrix GCS 3000 scanner (Affymetrix). For data extraction, Affymetrix GeneChip Command Console Software was used. Data were normalized by Robust Multi-array Average and detection above background methods using Expression Console 1.4.

### 2.6. Quantitative Real-Time PCR (qPCR)

Total RNA from 50 μL plasma and 200 μL CSF was isolated using miRNeasy serum/plasma kit (Qiagen), and total RNA from cells was isolated using miRNeasy micro kit (Qiagen) according to the manufacturer’s instruction. The cDNAs of miRNA and mRNA were synthesized using miScript RT II kit (Qiagen) and the PrimeScript™ RT Master Mix (Takara, Shiga, Japan), respectively. qPCR was conducted using miScript SYBR Green PCR Kit (Qiagen) for miRNAs and TB Green^®^ Premix Ex Taq™ (Takara) for mRNAs in LightCycler480 system (Roche, Basel, Switzerland). Primers for miRNAs were purchased from Qiagen. Primers for quantification of mRNAs are described in Table S4. The level of miRNAs in plasma and CSF samples was calculated using ΔCt method with reference miRNAs which were selected by referring to recommendation in Biofluids guidelines by Exiqon (Vedbaek, Denmark). The relative level of miRNAs and mRNAs in cells was calculated using ΔΔCt method with reference to the control group normalized by RNU6 for miRNAs and GAPDH for mRNAs.

### 2.7. Biotinylated-miRNA Pull-Down Assay

Biotinylated-miRNA pull-down assay was performed as described previously [31]. Briefly, H4-APPswe cells were transfected with 75 nM biotinylated-miR-1273g-3p or biotinylated cel-miR-39-3p as a negative control (Exiqon). After 24 h, cells were lysed with hypotonic buffer containing 10 mM KCl, 1.5 mM MgCl_2_, 10 mM Tris-HCl (pH 7.5), 5 mM dithiothreitol, 0.5% NP40, 50 U/mL SUPERaseIn (Invitrogen) and 1x protease inhibitor cocktail (Roche). The supernatants were transferred to 25 μL of streptavidin-containing myOne C1 Dynabeads (Invitrogen) which were pre-blocked with 1 μg/μL BSA and MS2 RNA (Roche), and the same volume of 2 M NaCl hypotonic buffer as the lysate was added. The mixture was incubated for 30 min with gentle rotation and the beads were washed with 1 M NaCl hypotonic buffer. Total RNA was extracted using miRNeasy micro kit (Qiagen) according to the manufacturer’s instruction.

### 2.8. RNA Sequencing (RNAseq) and Gene Ontology (GO) Analysis

A library of mRNAs was constructed using TruSeq Stranded Total RNA H/M/R Prep Kit (Illumina, San Diego, CA, USA) and the quality of library DNA was confirmed using 2200 TapeStation (Agilent). RNAseq was conducted using HiSeq 2500 system (Illumina). Sequence reads were mapped with human reference genome (hg19) using Tophat v2.0.13 with 79.64% of mapping rates and abundance of mRNAs represented by fragment per kilobase of transcript per million was estimated using Cuffdiff v. 2.2.0. GO was analyzed using DAVID Bioinformatics Resources v6.8 (https://david.abcc.ncifcrf.gov/, accessed on 5 January 2018 through 22 September 2021).

### 2.9. Bioinformatics Analysis

We analyzed the expression of GLRX5, MTCH1 and TIMM13 in the human prefrontal cortex by using RNAseq data for AaD patients (GN accession: GN368) and normal participants (GN accession: GN367) from the Harvard Brain Tissue Resource Center [32] provided in the publicly available GeneNetwork database (http://www.genenetwork.org/, accessed on 29 April 2021).

### 2.10. Measurements of Oxygen Consumption Rate (OCR)

After 24 h of transfection, cells were seeded on XFp Miniplate (Agilent) at a density of 20,000 cells/80 μL for H4-APPswe and 40,000 cells/80 μL for SH-SY5Y. After 24 h, media was changed to 180 μL of Seahorse XF DMEM (Agilent) supplemented with 10 mM glucose (Agilent), 1 mM sodium pyruvate (Agilent) and 1x Glutamax (Gibco). OCR was analyzed using Seahorse XFp analyzer (Agilent) with sequential addition of 1.5 μM Oligomycin, carbonyl cyaide-4-(trifluoromethoxy)phenylhydrazone (FCCP, 2 μM for H4-APPswe and 0.5 μM for SH-SY5Y) and 0.5 μM rotenone/antimycin A in Seahorse XFp Cell Mito Stress Test Kit (Agilent). The data were analyzed using WAVE software (Agilent) and normalized by cell viability.

### 2.11. Fluorescence Immunohistochemistry

Paraffin-sections were rehydrated and boiled in epitope retrieval solution (IHC World, Woodstock, MD, USA), followed by incubation with antibodies against TIMM13 (Novus, USA; NBP2-13431). Antigen-antibody complexes were visualized using the Alexa Fluor conjugated secondary antibody (Invitrogen). Aβ plaques were stained with 0.01 mg/mL Methoxy-X04 for 30 min. Fluorescence images were obtained using a Fluoview FV 1000 confocal microscope (Olympus, Japan) with FV10-MSASW software and analyzed using MetaMorph software (Molecular Devices, San Jose, CA, USA).

### 2.12. Neurite Outgrowth Analysis

SH-SY5Y cells were differentiated with retinoid acid and brain-derived neurotrophic factor as previously reported [33]. Differentiated cells were transfected with miR-1273g-3p mimic and negative control at day 14 and immunostained using anti-MAP2 antibody (Sigma, St. Louis, MO, USA). Fluorescence images were obtained using a Fluoview FV 1000 confocal microscope (Olympus, Japan) with FV10-MSASW software and analyzed using MetaMorph software (Molecular Devices, San Jose, CA, USA).

### 2.13. Statistical Analysis

Differences between two groups were assessed using Student’s *t*-test. To evaluate sensitivity and specificity of miR-1273g-3p for classification of each stage of AD group from control group, receiver operating characteristic (ROC) curve and the area under curve (AUC) were calculated. Correlation analysis between two groups was performed using Pearson’s correlation. Variability in all plots and graphs is presented as the ± SEM. All *p* < 0.05 were considered to be significant.

## 3. Results

### 3.1. miR-1273g-3p Is Elevated in the Plasma and CSF of AD Patients

**Figure 1:**
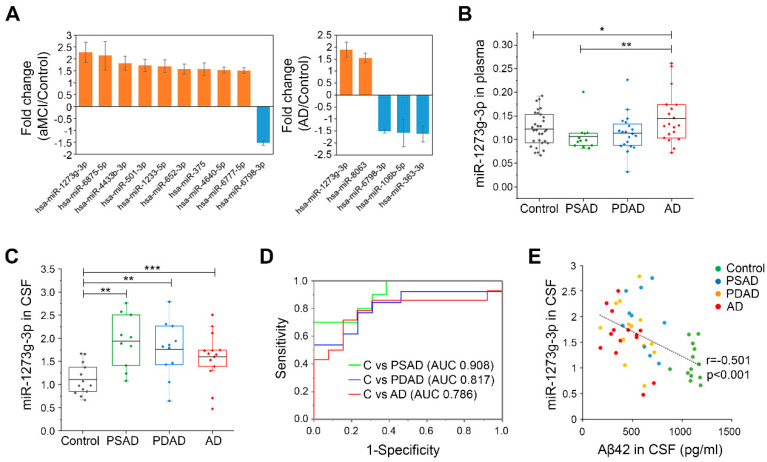
Analysis of miRNAs in plasma and CSF from four groups of AD. (**A**) Alzheimer’s disease (AD)-associated miRNAs selected by microarray analysis of miRNAs in plasma from 24 amnestic mild cognitive impairment (aMCI) patients, 36 AD patients, and 36 controls (absolute fold change > 1.5; *p* < 0.05, Student’s *t*-test). Bar graphs show fold change of miRNAs in aMCI (left) and AD (right) relative to the control. Orange and blue bars present up- and downregulated miRNAs, respectively. Data are presented as mean ± SEM. (**B**) Quantification of miR-1273g-3p in plasma from the presymptomatic AD (PSAD, n = 12), prodromal AD (PDAD, n = 20), AD (n = 20), and control (n = 31) groups, as assessed by qPCR. Column scatter plots present the level of miR-1273g-3p in individual samples. Each data point is the average value obtained from two or four independent analyses performed using the same plasma sample. The data were normalized by average Ct values of four control miRNAs (miR-191-5p, miR-451a, miR-425-5p, and miR-23a-3p). * *p* < 0.05, ** *p* < 0.01 (Student’s *t*-test). (**C**) Quantification of miR-1273g-3p in cerebrospinal fluid (CSF) from the PSAD (n = 10), PDAD (n = 13), AD (n = 14), and control (n = 13) groups, as assessed by qPCR. Column scatter plot presents the level of miR-1273g-3p for individual samples. Data were normalized by miR-23a-3p, which was the only utilized normalization-control miRNA detectable in CSF. ** *p* < 0.01, *** *p* < 0.001 (Student’s *t*-test). (**D**) Receiver operating characteristic (ROC) curves for control vs. PSAD (green line), control vs. PDAD (blue line), and control vs. AD (red line), as generated using qPCR data for miR-1273g-3p in CSF. The areas under the ROC curve (AUC) of each analysis are presented in the plot. (**E**) Correlation plot of the miR-1273g-3p level in CSF versus the Aβ42 level in CSF (n = 50). Data on the Aβ42 level in CSF were obtained from the National Research Center for Dementia (Chosun University, Gwangju, Korea). Significance of correlation was tested by Pearson’s correlation. The upper and lower limits of the box plot indicate the 75th and 25th percentiles, and the line in each box indicates the mean of each group. C, control.

To identify AD-associated miRNAs, we obtained plasma samples from 24 aMCI patients (pooled into 6 samples), 36 AD patients (pooled into 9 samples) and 36 age-matched cognitively normal individuals (controls, pooled into 9 samples) and screened miRNAs in these samples using a GeneChip^TM^ miRNA 4.0 array. The participants were at least 65 years of age who had been clinically diagnosed with AD but had not been subjected to verification of this diagnosis by amyloid-PET imaging or CSF test (Cohort 1 in Table 1). Microarray analysis of plasma samples allowed us to identify 10 miRNAs that were differentially expressed in aMCI patients versus controls and 5 miRNAs that were differentially expressed in AD patients versus controls. Among them, we focused on miR-1273g-3p that dramatically increased in plasma of both aMCI and AD patients (Figure 1A, Table S1). miR-1273g-3p was quantified by qPCR in individual plasma samples of a new cohort (n = 83, age ≥ 65 years), in which the participants had been precisely diagnosed with amyloid-PET imaging. We grouped the participants as follows: presymptomatic AD (PSAD; Aβ-positive but cognitively normal, n = 12), prodromal AD (PDAD; Aβ-positive aMCI, n = 20), AD (Aβ-positive dementia, n = 20), and control (Aβ-negative and cognitively normal, n = 31) (Cohort 2 in Table 1). Our analysis showed that miR-1273g-3p was significantly increased in the plasma of AD patients compared to PSAD and control individuals but there was no significant difference in this parameter between PDAD and control individuals (Figure 1B, Table S2).

To further substantiate the association of miR-1273g-3p with AD, we examined the levels of miR-1273g-3p in CSF of PSAD (n = 10), PDAD (n = 13), AD (n = 14), and control (n = 13) individuals using qPCR (Cohort 3 in Table 1). CSF samples were obtained from the same participants whose samples were used for qPCR analysis of plasma miRNAs, and were further obtained for seven new participants, including two controls and one PSAD, one PDAD, and three AD patients. Compared to controls, significant increase in the CSF level of miR-1273g-3p was detected in PSAD, PDAD, and AD patients (Figure 1C). In addition, the level of miR-1273g-3p in CSF was relatively higher than the levels of other miRNAs, such as AD-related miR-29a-3p [20], miR-16-5p and miR-451a, which are highly abundant in blood [34] (Table S3). The ROC curve generated using CSF-derived qPCR data indicated that the level of miR-1273g-3p could be used to clearly distinguish PSAD, PDAD and AD patients from age-matched controls (AUC > 0.75) (Figure 1D). Moreover, the level of CSF miR-1273g-3p was negatively correlated with the level of CSF Aβ42, which is known to decrease with AD progression [35] (Figure 1E). Based on these data, we suggest that miR-1273g-3p is an AD-associated miRNA.

Data are presented as mean ± standard deviation. K-MMSE, Korean mini-mental state exam; aMCI, amnestic mild cognitive impairment; PSAD, presymptomatic Alzheimer’s disease; PDAD, prodromal Alzheimer’s disease; AD, Alzheimer’s disease.

### 3.2. miR-1273g-3p Facilitates Aβ Production in an AD Model Cell Line

**Figure 2:**
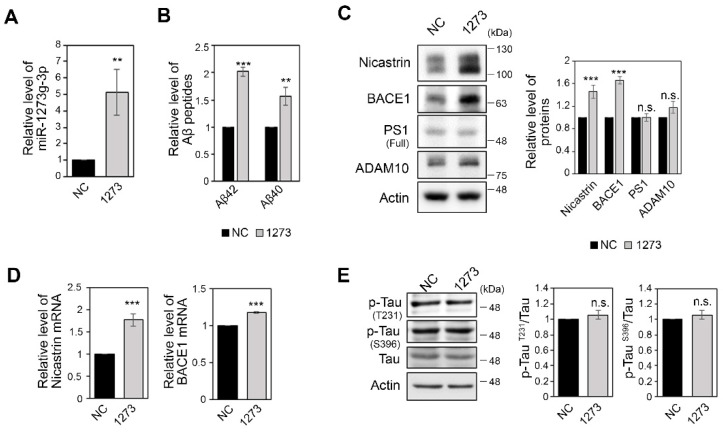
miR-1273g-3p increases Aβ production in H4-APPswe cells. (**A**) The level of miR-1273g-3p measured by qPCR in H4-APPswe cells transfected with miR-1273g-3p mimic relative to negative control cells. Data were normalized by RNU6 (n = 3). (**B**) Enzyme-linked immunosorbent assay (ELISA) of Aβ42 and Aβ40 in conditioned media of H4-APPswe cells transfected with miR-1273g-3p mimic or negative control (n = 3). (**C**) Western blotting assays of the levels of nicastrin, BACE1, PS1 and ADAM10 in H4-APPswe cells transfected with miR-1273g-3p mimic or negative control. Bar graphs show densitometric results for BACE1 and PS1. Data were normalized relative to expression of actin (n = 4). (**D**) Quantification of nicastrin and BACE1 mRNAs quantified by qPCR in H4-APPswe cells transfected with miR-1273g-3p mimic or negative control. Data were normalized relative to expression of GAPDH mRNA (n = 4). (**E**) Western blotting for p-Tau^T231^, p-Tau^S396^, and total-Tau in H4-APPswe cells transfected with miR-1273g-3p mimic or negative control. Densitometric results are presented as bar graphs. Actin was used as a loading control (n = 4). All data are presented as mean ± SEM. ** *p* < 0.01; *** *p* < 0.001; n.s., non-significant (Student’s *t*-test). NC, mimic negative control; 1273, miR-1273g-3p mimic.

To investigate the association of miR-1273g-3p with AD progression, we examined changes in Aβ production and hyper-phosphorylation of tau in neuroglioma H4 cells expressing APPswe^mut^; H4-APPswe), an AD model cell line. We overexpressed miR-1273g-3p by transfecting cells with a synthetic miR-1273g-3p mimic (Figure 2A). First, we quantified the concentration of Aβ42 and Aβ40 in conditioned medium of H4-APPswe cells by ELISA. Both Aβ42 and Aβ40 were considerably increased in conditioned medium of miR-1273g-3p-overexpressing cells compared to the negative control (Figure 2B). The increase in Aβ in conditioned medium of miR-1273g-3p overexpressing H4-APPswe was validated by Western blotting (Figure S1A). In addition, the level of APP protein was significantly higher, whereas the levels of c-terminal fragments (CTFs) were not changed, in miR-1273g-3p overexpressing H4-APPswe cells compared with negative control cells (Figure S1A).

Based on these observations, we tested the expression of APP-processing genes, such as BACE1, presenilin 1 (PS1), nicastrin and a disintegrin and metalloproteinase 10 (ADAM10). Western blot analysis demonstrated that the protein levels of BACE1 and nicastrin were significantly increased by miR-1273g-3p overexpression in H4-APPswe cells, whereas the levels of PS1 and ADAM10 were unchanged (Figure 2C). The mRNA levels of BACE1 and nicastrin were also increased, indicating that the expressions of BACE1 and nicastrin are transcriptionally regulated by miR-1273g-3p (Figure 2D). miR-1273g-3p-associated increases in BACE1 and Aβ42 production were observed even at lower levels of miR-1273g-3p overexpression which mimics the two-fold increases observed in the CSF of AD patients (Figure S1B–D). The expression levels of p-Tau^T231^ and p-Tau^S396^ were not altered by miR-1273g-3p overexpression in H4-APPswe cells (Figure 2E). Taken together, these results suggest that the increase of miR-1273g-3p expression induces Aβ production in H4-APPswe cells.

### 3.3. The miR-1273g-3p-Mediated Upregulation of BACE1 Is Caused by Oxidative Stress

**Figure 3:**
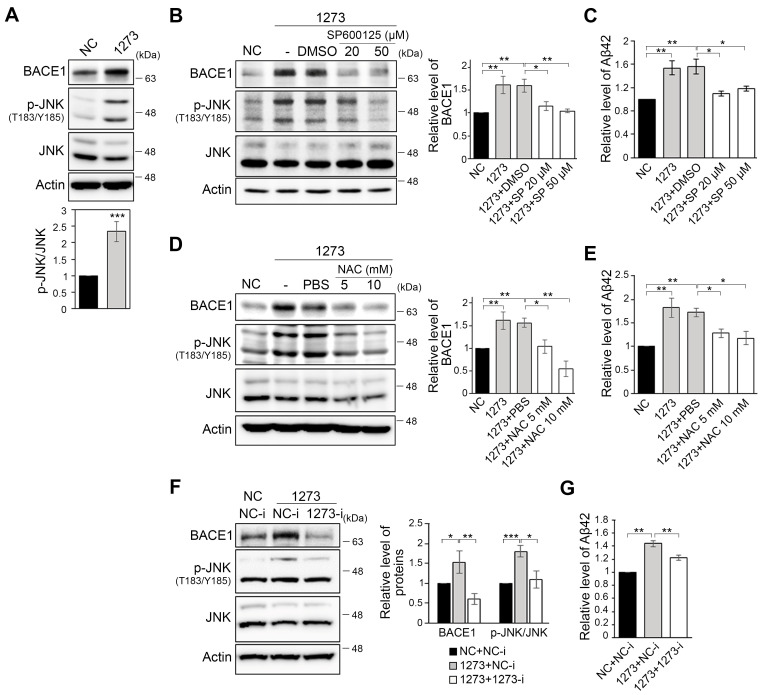
miR-1273g-3p induces oxidative stress to mediate the upregulation of BACE1 in H4-APPswe cells. (**A**) Western blotting for BACE1, p-JNK^T183/Y185^, and JNK in H4-APPswe cells transfected with miR-1273g-3p mimic or negative control. Ratio of p-JNK^T183/Y185^ to JNK is presented as a bar graph. Actin was used as a loading control (n = 5). (**B**,**C**) Inhibition of JNK activation in miR-1273g-3p-overexpressing H4-APPswe cells. SP600125 or DMSO were applied to the cells at 24 h post-transfection, and the cells were incubated for an additional 24 h. Cell lysates were analyzed by Western blotting against BACE1, p-JNK^T183/Y185^, and JNK (**B**) (n = 4), and the conditioned media were analyzed for amyloid beta (Aβ) 42 by ELISA (**C**) (n = 3). (**D**,**E**) Treatment of the antioxidant, N-acetylcysteine (NAC), to H4-APPswe cells overexpressing miR-1273g-3p. NAC or PBS was treated to cells at 24 h post-transfection and the cells were incubated for an additional 24 h. BACE1 expression was analyzed by Western blotting. p-JNK^T183/Y185^ and JNK were detected to check the decrease of oxidative stress under NAC treatment (**D**). Aβ42 concentration in conditioned media was analyzed by ELISA (**E**) (n = 3 per experiment). (**F**,**G**) Inhibition of miR-1273g-3p in miR-1273g-3p-overexpressing H4-APPswe cells co-transfected with miR-1273g-3p mimic and inhibitor. BACE1, p-JNK^T183/Y185^, and JNK in cell lysates were quantified by Western blotting (**F**), and the Aβ42 concentration in conditioned media was analyzed by ELISA (**G**) (n = 3 per experiment). All data are presented as mean ± SEM. * *p* < 0.05, ** *p* < 0.01, *** *p* < 0.001 (Student’s *t*-test). NC, mimic negative control; 1273, miR-1273g-3p mimic; NC-i, inhibitor negative control; 1273-i, miR-1273g-3p inhibitor; SP, SP600125.

As BACE1 is the rate-limiting enzyme for production of Aβ peptides [36], we first investigated the factors that regulate BACE1 expression. BACE1 expression is well known to be regulated by stress-induced molecules, such as hypoxia induced factor 1α (HIF1α) and c-Jun N-terminal kinase (JNK) [16,18,37]. Thus, to elucidate the mechanism by which miR-1273g-3p upregulates BACE1 and downstream Aβ production, we examined the ability of miR-1273g-3p overexpression to alter one of the stress-induced molecule, JNK. The overexpression of miR-1273g-3p in H4-APPswe cells dramatically increased the level of p-JNK^T183/Y185^ relative to JNK (Figure 3A). In addition, treatment of miR-1273g-3p-overexpressing H4-APPswe cells with the JNK inhibitor, SP600125 (Figure 3B), or the antioxidant, N-acetylcysteine (NAC) (Figure 3D), decreased the levels of BACE1 and p-JNK^T183/Y185^ back to the basal level seen in the negative control. The increased level of Aβ42 in the conditioned medium was also reduced to the basal level by both treatments (Figure 3C,E). In contrast, the levels of APP and nicastrin were not significantly reduced by treatment with SP600125 or NAC (Figure S2A,B), indicating that miR-1273g-3p induced overproduction of Aβ may be due primarily to the increase of BACE1 expression caused by oxidative stress. The treatment of mimic transfected H4-APPswe cells with an miR-1273g-3p inhibitor effectively restored BACE1 and p-JNK^T183/Y185^ to their basal levels (Figure 3F) and slightly reduced the level of Aβ42 in the conditioned medium (Figure 3G). Collectively, these data support the notion that miR-1273g-3p overexpression upregulates BACE1 expression via JNK signaling, and thereby induces Aβ42 production. 

### 3.4. miR-1273g-3p Impairs Mitochondrial Function in H4-APPswe and SH-SY5Y Cells

**Figure 4:**
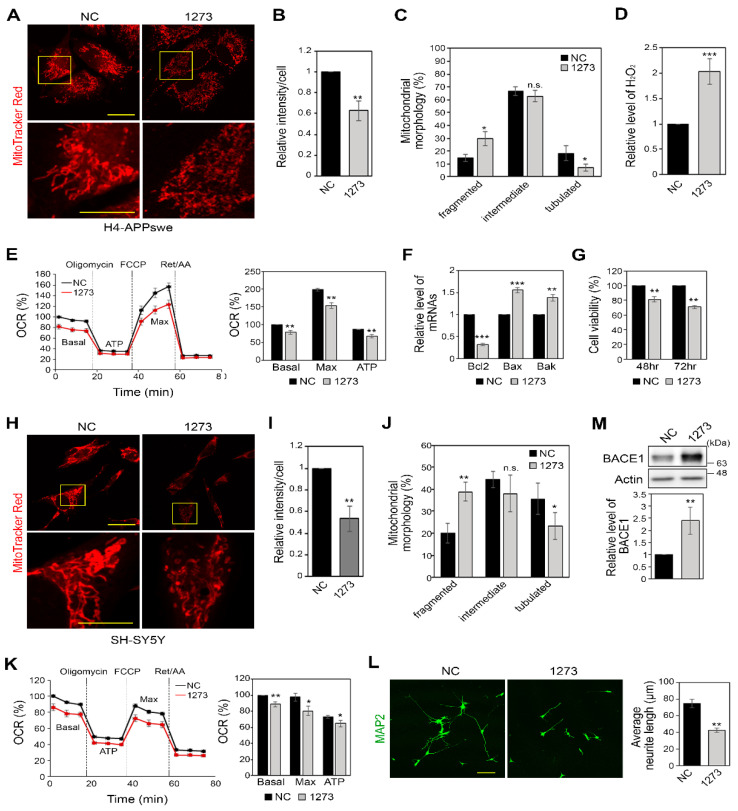
miR-1273g-3p impairs mitochondrial function in H4-APPswe and SH-SY5Y cells. (**A**–**C**) Representative images of MitoTracker Red CMX Ros (MitoTracker Red) staining for H4-APPswe cells transfected with miR-1273g-3p mimic or negative control. Magnified views of the boxed area are presented in the second row. Scale bar: first row, 20 μm; second row, 10 μm (**A**). Intensity of MitoTracker Red is shown in (**B**) (n = 3, 26-65 cells were analyzed per experiment) and analysis of mitochondrial morphological changes is shown in (**C**) (n = 3, 43-135 cells per experiment). (**D**–**G**) Relative concentration of H_2_O_2_ (**D**) (n = 3), oxygen consumption rate (OCR) analysis (**E**) (n = 4), quantification of Bcl-2, Bax, and Bak mRNAs by qPCR (F) (n = 4), and percentage of cell viability assessed by WST-1 (**G**) (n = 3) in H4-APPswe cells transfected with miR-1273g-3p mimic or negative control. (**H**–**J**) Representative images of MitoTracker Red staining for SH-SY5Y cells transfected with miR-1273g-3p mimic or negative control. Magnified views of the boxed area are presented in the second row. Scale bar: first row, 20 μm; second row, 10 μm (**H**). Intensity of MitoTracker Red is shown in (**I**) (n = 3, 42-137 cells per experiment) and analysis of mitochondrial morphological changes is shown in (**J**) (n = 3, 57-131 cells per experiment). (**K**–**M**) Measurements of OCR (**K**) (n = 4), neurite outgrowth analysis by immunostaining of MAP2 (**L**) (n = 3; Scale bar: 100 μm), and Western blotting for BACE1 (**M**) (n = 3) in SH-SY5Y cells transfected with miR-1273g-3p mimic or negative control. Mitochondrial morphology was defined as fragmented, mainly round shape; tubulated, mainly long shape; and intermediate, mixture of round and tubulated shape. Real-time OCR data are presented as a percentage relative to the first negative control measurement; bar graph indicates the percentage of OCR relative to the basal value of the negative control. All data are presented as mean ± SEM. * *p* < 0.05; ** *p* < 0.01; *** *p* < 0.001; n.s., non-significant (Student’s *t*-test). NC, mimic negative control; 1273, miR-1273g-3p mimic.

As oxidative stress is caused by mitochondrial dysfunction [7], we next investigated the changes of mitochondrial function under miR-1273g-3p overexpression. When H4-APPswe cells overexpressing miR-1273g-3p and negative control cells were stained with MitoTracker^TM^ Red CMX Ros, the cells overexpressing miR-1273g-3p showed significantly lower staining intensity compared to negative control cells (Figure 4A,B), suggesting that mitochondrial membrane potential was defected in the former. This result was verified by staining with the carbocyanine-based reagent MitoTracker^TM^ Deep Red FM (Figure S3A). In addition, miR-1273g-3p-overexpressing cells demonstrated dramatic morphological changes of mitochondria. The numbers of cells with fragmented mitochondria were increased and the numbers of tubulated mitochondria decreased (Figure 4C). These findings supported the qPCR results showing that the levels of expression of MFN1/2 and OPA1, which participate in mitochondrial fusion [38], were decreased, whereas the level of expression of FIS1, which facilitates mitochondrial fission [39], was increased in miR-1273g-3p overexpressing H4-APPswe cells (Figure S3B). The increased concentration of cellular hydrogen peroxide (H_2_O_2_) and the intensity of CellROX^TM^ Deep Red staining, which detects intracellular ROS, in miR-1273g-3p overexpressing H4-APPswe cells indicate that these cells are under oxidative stress (Figure 4D and Figure S3C). When we measured the levels of mRNAs encoding NRF2, SOD1/2 and ACO2, which are responsible for anti-oxidative stress, we found that miR-1273g-3p overexpression significantly decreased the levels of SOD1/2 (Figure S3D).

In analysis of the real-time OCR, basal and ATP-linked OCR were considerably decreased in miR-1273g-3p-transfected H4-APPswe cells. The maximum OCR, which was measured using the uncoupling reagent, FCCP, was also significantly reduced by miR-1273g-3p (Figure 4E). The glycolysis rate, as determined by measuring the extracellular acidification rate (ECAR) while measuring OCR, was also significantly decreased in miR-1273g-3p-overexpressing H4-APPswe cells (Figure S3E), indicating that these cells were energy deprived. In miR-1273g-3p mimic-transfected H4-APPswe cells, the anti-apoptotic gene, Bcl-2, was decreased; the pro-apoptotic genes, Bax and Bak, were significantly increased (Figure 4F); and cell viability was reduced (Figure 4G).

To elucidate the function of miR-1273g-3p in neuronal cells, we analyzed the changes of mitochondrial function and BACE1 expression using neuroblastoma cell line SH-SY5Y. In miR-1273g-3p-overexpressing SH-SY5Y cells stained with MitoTracker^TM^ Red CMX Ros, the fluorescence intensity was decreased, the number of cells with fragmented mitochondria was considerably increased, and the number of cells with tubulated mitochondria was decreased (Figure 4H–J), reflecting a loss of mitochondrial membrane potential. The basal and maximal OCR were decreased (Figure 4K) and ECAR was slightly, but not significantly, decreased (Figure S3F) in miR-1273g-3p-overexpressing SH-SY5Y cells, indicating that ATP production and the mitochondrial respiration capacity were reduced in these cells. This was associated with an inhibition of neurite outgrowth (Figure 4L) and increased BACE1 expression (Figure 4M). Taken together, our results indicate that miR-1273g-3p is likely to induce mitochondrial dysfunction, and therefore an abnormal increase of miR-1273g-3p would disrupt mitochondrial functions leading to energy deprivation, oxidative stress, and eventual cell degeneration. 

### 3.5. miR-1273g-3p Interacts with and Downregulates the Expression Levels of Mitochondrial Genes in H4-APPswe Cells

**Figure 5:**
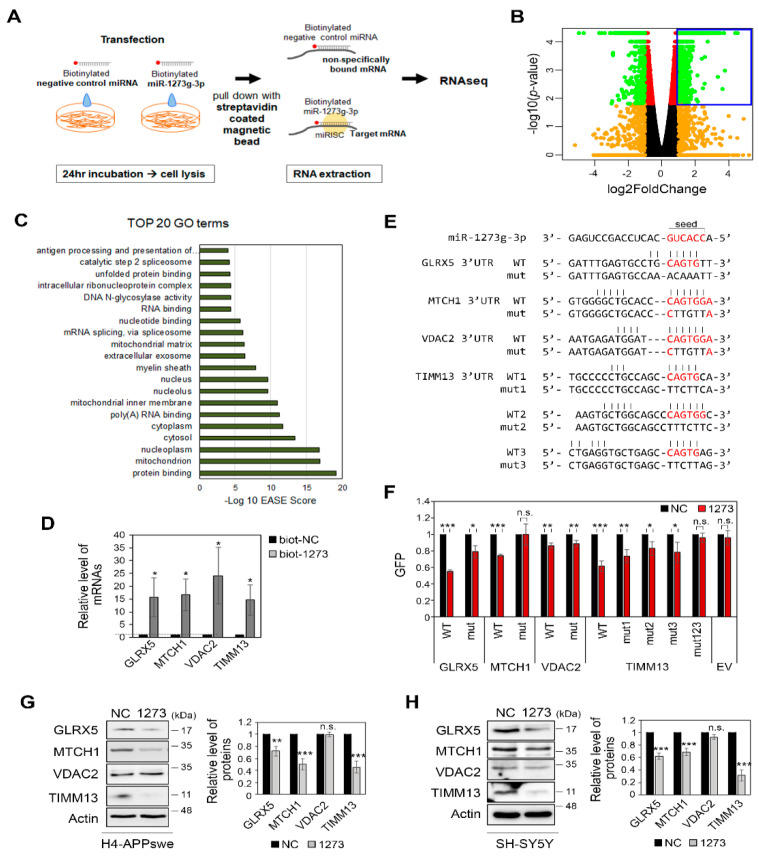
Identification of target genes for miR-1273g-3p. (**A**) Experimental scheme of pull-down assays performed using biotinylated-miR-1273g-3p or negative control (miR-cel-39-3p) in H4-APPswe cells. mRNAs interacting with biotinylated-miR-1273g-3p or negative control were pulled down by streptavidin beads and analyzed by high-throughput RNA sequencing (RNAseq). (**B**) Volcano plot indicates the log_2_fold change versus the –log_10_ *p*-value of the genes that interacted more strongly with biotinylated-miR-1273g-3p relative to the negative control. Blue box indicates 1539 genes whose mRNAs were significantly enriched in pull-down samples of biotinylated-miR-1273g-3p (n = 3, fold change > 2, *p* > 0.05, Student’s *t*-test). (**C**) Top 20 Gene Ontology (GO) terms of the 1539 gene transcripts that interacted with biotinylated-miR-1273g-3p. Bar graph presents the –log10 EASE scores. (**D**) Validation that the top 4 genes among 192 mitochondrial genes interacted with biotinylated-miR-1273g-3p, as assessed by qPCR analysis of the pull-down samples. Bar graph shows the level of each mRNA relative to the negative control. Data were normalized by GAPDH in the supernatant of each pull-down sample (n = 4). (**E**) The putative target sequences of miR-1273g-3p in 3′UTRs of GLRX5, MTCH1, VDAC2 and TIMM13. Sequences in 3′UTR of each gene complementary to the seed sequence of miR-1273g-3p (red) were mutated as indicated. (**F**) Reporter gene assays were performed using WT or mutant GFP reporter vector with the 3′UTRs of GLRX5, MTCH1, VDAC2, and TIMM13. Bar graph shows densitometric results of GFP normalized by actin (n = 3). (**G**,**H**) Western blotting for GLRX5, MTCH1, VDAC2, and TIMM13 in H4-APPswe cells (**G**) (n = 6) and SH-SY5Y cells (**H**) (n = 5) transfected with miR-1273g-3p mimic or negative control. All data are presented as mean ± SEM. * *p* < 0.05, ** *p* < 0.01, *** *p* < 0.001 (Student’s *t*-test). biot-NC, biotinylated-negative control; biot-1273, biotinylated-miR-1273g-3p; NC, mimic negative control; 1273, miR-1273g-3p mimic; EV, empty vector; mut, mutant.

To identify the target genes of miR-1273g-3p, we pulled down mRNAs with biotinylated-miR-1273g-3p (biot-miR-1273g-3p) in H4-APPswe cells and profiled the mRNAs by high-throughput RNAseq (Figure 5A). After pull-down of biot-miR-1273g-3p, we analyzed the level of miR-1273g-3p and the reference miRNAs, miR-191-5p and miR-16-5p, in the pull-down samples and supernatants by qPCR to confirm the efficiency and specificity of the experiment (Figure S4A). RNAseq analysis of the pull-down samples identified 1539 genes whose mRNAs interacted with biot-miR-1273g-3p (Figure 5B). GO analysis predicted ‘mitochondrion’ as the most enriched GO term, with 192 genes falling into this category (Figure 5C). From among them, we used the p-values to select four mitochondrial genes for further analysis: GLRX5 (glutaredoxin 5), MTCH1 (mitochondrial carrier 1), VDAC2 (voltage dependent anion channel 2) and TIMM13 (translocase of inner mitochondrial membrane 13). We verified their binding with miR-1273g-3p by qPCR analysis (Figure 5D). 

To confirm that miR-1273g-3p modulates the expression levels of these target genes by binding directly to their 3′UTRs, we constructed reporter vectors in which the 3′UTR sequences of GLRX5, MTCH1, VDAC2, and TIMM13 were inserted after the stop codon of the GFP gene. TargetScan showed that the sequences of the 3′UTRs of MTCH1 and VDAC2 completely match the seed sequence of miR-1273g-3p, whereas GLRX5 and TIMM13 have one and three putative sequences, respectively, with partially matched bases. Each putative target sequence was replaced with mismatched bases in mutant constructs (Figure 5E). These reporter plasmids were co-transfected with miR-1273g-3p mimic or negative control into H4-APPswe cells. The expression of GFP by the reporter vectors containing the WT 3′UTR sequences of GLRX5, MTCH1 and TIMM13 were markedly downregulated in the presence of miR-1273g-3p, whereas the expression of GFP by reporter vector containing the WT 3′UTR of VDAC2 was not (Figure 5F and Figure S4B). The miR-1273g-3p-mediated downregulation of GFP was completely abrogated by a mutation in the 3′UTR of MTCH1 and by a triple mutation in the 3′UTR of TIMM13, and was partially abrogated by a mutation in the 3′UTR of GLRX5 and by single mutations in the 3′UTR of TIMM13 (Figure 5F and Figure S4B).

When miR-1273g-3p was overexpressed in H4-APPswe and SH-SY5Y cells, the protein expression levels of TIMM13, GLRX5, and MTCH1 were reduced whereas that of VDAC2 remained unchanged (Figure 5G,H). Cell staining further confirmed the reduction of TIMM13 and GLRX5 expression by miR-1273g-3p in H4-APPswe (Figure S4C). Transfection of various concentrations of miR-1273g-3p mimic into H4-APPswe cells dose-dependently downregulated TIMM13, GLRX5, and MTCH1 expression and increased JNK activation (Figure S4D). These data support that miR-1273g-3p interacts with and negatively regulates various mitochondrial genes in both H4-APPswe and SH-SY5Y cells. 

### 3.6. Modulation of the Expression of miR-1273g-3p Target Genes Affects Mitochondrial Function and Aβ42 Production

**Figure 6:**
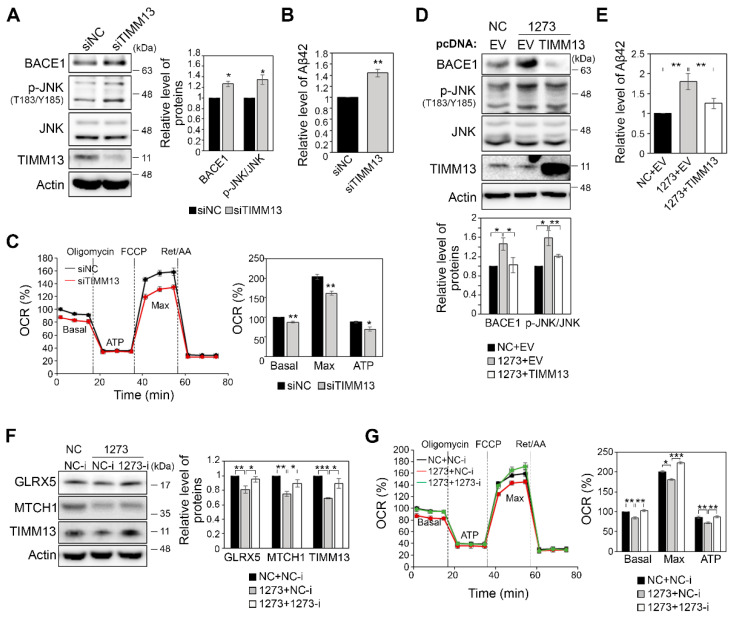
Mitochondrial function and Aβ42 production are altered by modulating the expression of miR-1273g-3p target genes. (**A**,**B**) TIMM13-targeting or negative control siRNAs were transfected into H4-APPswe cells, and cultures were subjected to Western blotting for BACE1, p-JNK^T183/Y185^, JNK, and TIMM13 in cell lysates (**A**) and ELISA for Aβ42 in conditioned media (**B**) (n = 3 per experiment). (**C**) Oxygen consumption rate (OCR) analysis of H4-APPswe cells transfected with siTIMM13 or negative control (n = 3, all experiments were performed in triplicate). (**D**,**E**) H4-APPswe cells were co-transfected with miR-1273g-3p mimic or negative control and pcDNA3.0-TIMM13 or empty vector. Cells were harvested for Western blotting of BACE1, p-JNK^T183/Y185^, JNK, and TIMM13 (**D**), and conditioned media were harvested for Aβ42 ELISA (**E**) (n = 3 per experiment). (**F**) H4-APPswe cells were co-transfected with miR-1273g-3p mimic and inhibitor, and Western blotting was performed for GLRX5, MTCH1, and TIMM13 after inhibition of miR-1273g-3p in miR-1273g-3p-overexpressing cells (n = 3). (**G**) OCR analysis of H4-APPswe cells co-transfected with miR-1273g-3p mimic and inhibitor or their negative controls (n = 3, all experiments were performed in triplicate). Real-time OCR data are presented as a percentage relative to the first negative control measurement. Bar graph indicates the percentage of OCR relative to the basal value of the negative control. All data are presented as mean ± SEM. * *p* < 0.05, ** *p* < 0.01, *** *p* < 0.001 (Student’s *t*-test). NC, mimic negative control; 1273, miR-1273g-3p mimic; EV, empty vector.

To further investigate the function of TIMM13, which was significantly downregulated in miR-1273g-3p-transfected H4-APPswe and SH-SY5Y cells, we used siRNA to knock down TIMM13 in H4-APPswe cells. We found that TIMM13 knockdown moderately increased the levels of BACE1, p-JNK, and Aβ42 (Figure 6A,B), but reduced the maximum OCR (Figure 6C). Based on these data, we sought to overexpress TIMM13 in miR-1273g-3p-overexpressing H4-APPswe cells, in which TIMM13 expression was downregulated. Indeed, we found that the overexpression of TIMM13 moderately restored BACE1 and Aβ42 production to the basal level in miR-1273g-3p-overexpressing H4-APPswe cells (Figure 6D,E). The activation of JNK signaling by miR-1273g-3p was also relieved by the overexpression of TIMM13 (Figure 6D). As we had found that treatment with an miR-1273g-3p inhibitor alleviated the increase of Aβ42 production in miR-1273g-3p-overexpressing H4-APPswe (see Figure 3F,G), we investigated whether the expression of target genes could be rescued by co-transfecting the miR-1273g-3p mimic and inhibitor into H4-APPswe cells. Western blot analyses revealed that the expression of GLRX5, MTCH1 and TIMM13, which were decreased by miR-1273g-3p overexpression, was recovered by miR-1273g-3p inhibitor in H4-APPswe cells (Figure 6F). The maximum and ATP-linked OCR were also restored to the level seen in the negative control by co-transfection of the miR-1273g-3p inhibitor in H4-APPswe cells (Figure 6G). Taken together, these data suggest that miR-1273g-3p regulates the expression levels of genes associated with mitochondrial function and in turn induces Aβ42 production.

### 3.7. TIMM13 Is Downregulated in Hippocampi of Human AD Patients

**Figure 7:**
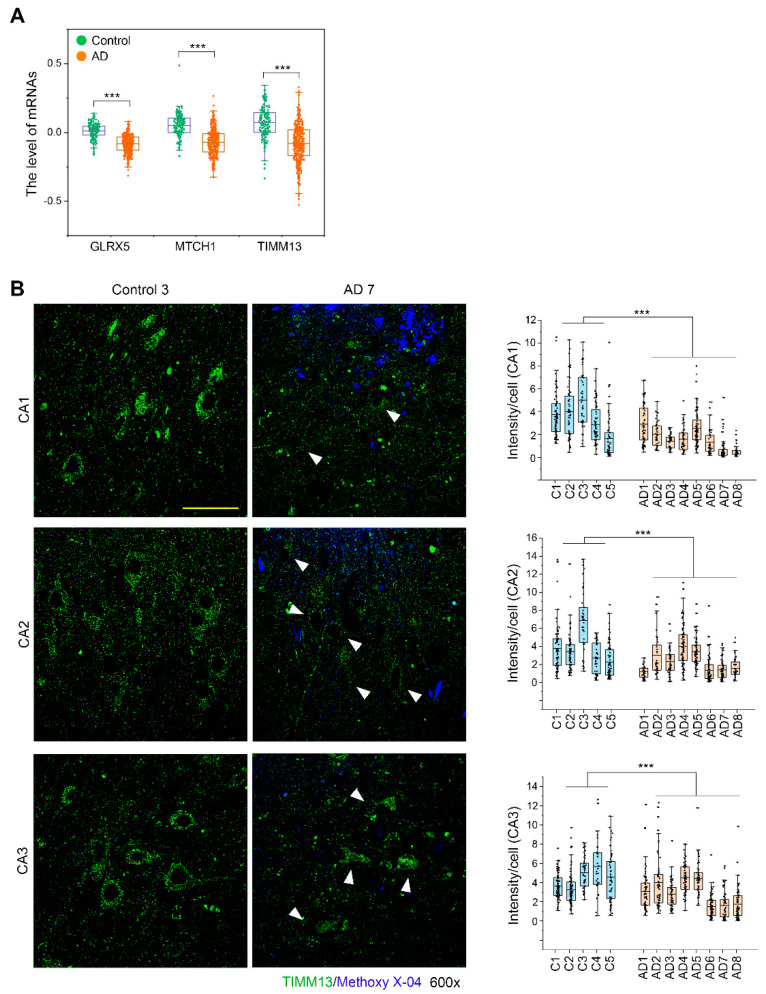
TIMM13 is downregulated in hippocampi of AD patients. (**A**) Expression levels of GLRX5, MTCH1, and TIMM13 mRNAs in human prefrontal cortex of AD patients (GN368, n = 383) and controls (GN367, n = 165). (**B**) Fluorescence immunohistochemistry of TIMM13 (green) with methoxy-X04 staining (blue) in human post-mortem hippocampal tissues from 8 AD patients and 5 controls. Representative images of the stratum pyramidal layer of CA1, CA2, and CA3 are shown. Arrows in images of AD patients indicate pyramidal neurons. Column scatter plots present the intensity of TIMM13 staining per each pyramidal neuron. The upper and lower limits of the box plot indicate the 75th and 25th percentiles; the line in each box indicates the average of each group; and whiskers indicate outliers. Scale bar: 50 μm. *** *p* < 0.001; n.s., non-significant (Student’s *t*-test).

We analyzed the expression levels of three miR-1273g-3p target genes, GLRX5, MTCH1, and TIMM13, in brain tissues from AD patients using the GN367 and GN368 datasets of GeneNetwork (http://www.genenetwork.org/, accessed 29 April 2021). The expression levels of three genes were significantly decreased in AD patients compared to controls (Figure 7A). To confirm these findings, we analyzed TIMM13 expression by fluorescence immunohistochemistry in human hippocampus tissues obtained from 8 AD patients and 5 controls. TIMM13 was abundantly expressed in normal brain, but was only faintly detected in the stratum pyramidal layers CA1, CA2 and CA3 of the hippocampus in AD brains (Figure 7B). Collectively, these data suggest that TIMM13 downregulation in AD brains is correlated with the pathogenesis of AD.

## 4. Discussion

Most AD patients have sporadic AD, consisting of late onset AD that begins after age 65 years, rather than familial AD. As the cause of sporadic AD has not yet been fully elucidated, it remains difficult to predict AD onset at early stage. This is a problem, because early diagnosis and treatment can delay AD progression [40]. Many studies have suggested that genetic mutations, such as those in APOE or TREM2, are closely associated with sporadic AD [41]. Epigenetic factors, such as DNA methylation, histone modification and non-coding RNAs, have been also suggested as risk factors of sporadic AD [42]. Owing to their essential role in fine-tuning gene expression, non-coding RNAs, particularly miRNAs, may be biomarkers of sporadic AD or even targets for treatment. miR-29a/b-1 clusters and miR-132-3p, which regulate the expression of genes involved in APP processing and tau phosphorylation, were shown to be differentially expressed in postmortem brains of AD patients [20,21]. To determine early miRNA markers, several studies profiled miRNAs in CSF or plasma from mild late onset AD or MCI patients [27,43]. However, miRNAs are not yet considered to be reliable biomarkers for AD diagnosis because they have not yet been sufficiently analyzed in the early stage of AD, nor have functional analyses been performed in this context. Using high-throughput analysis, the present study found that miR-1273g-3p is a novel AD-associated miRNA present in the plasma and CSF of individuals with PSAD who were presumed to be in the early stages of AD. We then elucidated its cellular function in AD progression.

We found that miR-1273g-3p was constantly elevated in CSF of PSAD, PDAD, and AD patients compared to controls, and also showed significantly higher levels in plasma of AD patients (Figure 1). This finding is supported by a study showing that the level of mir-1273g, a precursor of miR-1273g-3p, is one of miRNAs increased in blood from AD patients [44]. To our knowledge, however, no study has investigated the role of miR-1273g-3p in AD pathogenesis. In addition, our results are considered reliable due to the use of accurate AD diagnosis through amyloid-PET imaging and assessment of AD risk factors, and reproducibility across the three cohorts. We suggest that CSF miR-1273g-3p could be a good biomarker for early diagnosis of AD. However, we would need to establish a standard protocol for normalizing miRNA levels in plasma and CSF, prior to using miR-1273g-3p as a biomarker for clinical diagnosis. Also, longitudinal observation should be conducted to determine whether PSAD and/or PDAD patients progress to AD, as this could be a key to accurately diagnose cognitively normal but Aβ-positive people as potential AD patients. 

We used a pull-down assay to determine the function of miR-1273g-3p, and identified 1539 miR-1273g-3p-interacting transcripts with significant binding values in H4-APPswe cells. GO analysis predicted that this gene set is primarily involved in mitochondrial function. Among the 192 transcripts of mitochondrial genes found to interact with miR-1273g-3p, we confirmed that functional interactions occurred with GLRX5, MTCH1 and TIMM13 (Figure 5). In both H4-APPswe and SH-SY5Y cells, miR-1273g-3p expression had the greatest decreasing effect on TIMM13, which is known to import and insert certain proteins into the mitochondrial inner membrane, such as SLC25A12 and TIMM23 in complex with TIMM8 [45,46]. The mechanisms by which TIMM13 regulates mitochondrial function have not been fully elucidated. However, it is known that the neurodegenerative disease, Mohr–Tranebjaerg syndrome, occurs due to a mutation in TIMM8A, which acts as a complex with TIMM13, suggesting its association with neurodegenerative disease [47]. Knockdown of TIMM13 alone was associated with significant increases in JNK activation, BACE1 expression, and Aβ production and TIMM13 expression tended to be downregulated in the post-mortem AD brain. Overexpression of TIMM13 and inhibition of miR-1273g-3p in miR-1273g-3p-transfected cells restored the increased BACE1 expression and Aβ production to basal levels. These observations suggested that enhanced mitochondrial function could be helpful to prevent AD progression. This finding is supported by a previous report that the upregulation of various mitochondrial genes responsible for the mitochondrial stress response could rescue Aβ toxicity in AD model cell lines and AD model animals, including mouse and *C. elegans* [48]. Among the genes that interact with miR-1273g-3p, genes with the GO terms “RNA binding”, “RNA splicing” and “poly(A) RNA binding”, which show high significance, should not be overlooked to more fully understand the function of miR-1273g-3p. RNA binding proteins are essential for the regulation of gene expression through various RNA processing pathways, such as alternative polyadenylation (APA). In particular, the untranslated region (UTR)-APA has a definite impact on the function of miRNAs [49]. Among the miR-1273g-3p-interacting genes associated with RNA processing, RPL23 is a ribosomal protein that is a component of the 60S subunit, and PIN4 encodes a ribosomal RNA processing factor [50]. Negative regulation of these genes could affect the stability of mRNAs encoded by other genes targeted by miR-1273g-3p, indicating that the regulation of gene expression by miR-1273g-3p could affect its self-regulation. We found that, although VDAC2 significantly interacted with miR-1273g-3p, the expression of VDAC2 was unaffected by miR-1273g-3p overexpression. This unexpected result may have been due to the change in expression of factors that participate in RNA processing pathways, or to other regulatory factors. Although the functions of these miR-1273g-3p-interacting genes were not fully elucidated in the present study, we speculate that their changes may contribute to AD onset by triggering irreversible mitochondrial dysfunction and consequent cell degeneration.

Accumulated evidences indicate that mitochondrial dysfunction might be a primary factor in AD development [5,16,17]. Various factors have been proposed to induce mitochondrial dysfunction in AD progression. In particular, Aβ is reported to accumulate and inhibit peptide turnover in mitochondria, activate mitochondrial fission proteins, and eventually induce apoptosis [51,52,53]. In addition, defects in calcium signaling or neuroinflammation can impair mitochondrial function in AD [54,55]. However, the mechanism impairing mitochondria in AD have not yet been clearly identified, and AD remains a complex, multifactorial, and irreversible disease. Many recent studies have reported that various miRNAs can regulate mitochondrial activities in AD. For example, miR-34a, which is enhanced in brains of AD patients, targets mitochondrial genes (e.g., NDUFC2 and UQCRB) to reduce ATP production and glycolytic capacity [56]. miR-195 is elevated in hippocampus and targets mitofusin2, and its overexpression triggered the loss of mitochondrial membrane potential in the senescence-accelerated mouse-prone 8 mouse model [57]. Interestingly, miR-375, the level of which was increased in aMCI patients, as shown by our microarray data, was also reported to regulate mitochondrial function. The expression of miR-375 was increased in Aβ-treated SH-SY5Y cells and AD patients, and the inhibition of its expression alleviated oxidative stress injury and the apoptosis of Aβ-treated SH-SY5Y cells [58]. In addition, miR-375 promoted mitochondrial dependent apoptosis in patients with Stevens-Johnson syndrome and toxic epidermal necrolysis [59] and reduced insulin secretion in response to glucose and oxygen consumption related to glycolysis and pyruvate metabolism in rat and human islet cells [60]. Here, we report that miR-1273g-3p is increased in AD, and that increased miR-1273g-3p induces mitochondrial dysfunction to affect Aβ production. The previous and present findings therefore collectively suggest that the accumulation of dysregulated miRNAs caused by various stimuli might negatively affect homeostasis in brain and thereby cause irreversible brain degeneration.

A limitation of the present study is that the altered expression of miR-1273g-3p has not been directly confirmed in brain tissues of AD patients. The level of miR-1273g-3p has been reported to be increased in human umbilical vein endothelial cells (HUVECs) stressed by acute glucose fluctuation [61] and in the LoVo colorectal cancer cell line [62]. miR-1273g-3p can also affect megakaryocyte differentiation by regulating cyclin and cyclin dependent kinases [63]. Although analysis of the TissueAtlas database (https://ccb-web.cs.uni-saarland.de/tissueatlas/, accessed 10 August 2021) predicted that miR-1273g-3p is highly expressed in brain tissue, no experimental evidence to date has shown that miR-1273g-3p is expressed in brain cells, and we were unable to identify the cells that express and secrete miR-1273g-3p into the CSF and blood of AD patients. Nevertheless, this study showed that in AD patients miR-1273g-3p is significantly increased in CSF, which is in direct contact with brain parenchyma, as well as showing the functions of miR-1273g-3p in two types of brain cell line, H4-APPswe (neuroglioma) and SH-SY5Y (neuroblastoma) cells. miRNAs can regulate gene expression not only in cells that express miRNAs but in other cells by secretion and uptake [64]. These findings suggest that miR-1273g-3p could affect various brain-derived cell types via paracrine or endocrine as well as autocrine effects.

In conclusion, we herein report that miR-1273g-3p is increased at an early stage of AD, and that this AD-related upregulation of miR-1273g-3p induced mitochondrial dysfunction by targeting various mitochondrial genes to facilitate Aβ production and neurodegeneration. Thus, miR-1273g-3p might be a biomarker for early diagnosis of AD and a potential therapeutic target for AD progression.

## Figures and Tables

**Table 1:** Clinical and demographic data of participants.

Cohort	Diagnosis	n (M/F)	Age, y	*p*-Value	K-MMSE	*p*-Value	Amyloid PET
Microarray for miRNAs in plasma (Cohort 1)	Control	36 (16/20)	72.86 ± 4.72	-	27.31 ± 2.69	-	-
	aMCI	24 (12/12)	74.22 ± 4.51	0.115	25.94 ± 2.54	0.018	-
	AD	36 (20/16)	73.58 ± 5.72	0.344	17.34 ± 8.17	2.35 × 10^−9^	-
qPCR for miRNAs in plasma (Cohort 2)	Control	31 (18/13)	72.85 ± 5.31	-	27.10 ± 1.83		negative
	PSAD	12 (6/6)	73.99 ± 3.32	0.247	26.67 ± 3.55	0.28	positive
	PDAD	20 (10/10)	74.38 ± 4.80	0.150	25.35 ± 3.69	1.45 × 10^−2^	positive
	AD	20 (8/12)	72.54 ± 3.61	0.411	17.50 ± 4.83	8.67 × 10^−14^	positive
qPCR for miRNAs in CSF (Cohort 3)	Control	13 (7/6)	74.46 ± 4.66		26.62 ± 1.56		negative
	PSAD	10 (5/5)	73.75 ± 4.24	0.373	26.90 ± 3.28	0.393	positive
	PDAD	13 (7/6)	74.08 ± 5.87	0.428	25.46 ± 4.03	0.173	positive
	AD	14 (7/7)	70.39 ± 4.82	0.017	17.93 ± 4.91	1.13 × 10^−6^	positive

## Data Availability

The data that support the findings of this study are available from the corresponding author upon reasonable request.

## Supplementary Materials

The following are available online at https://www.mdpi.com/article/10.3390/cells10102697/s1, Figure S1 (related to Figure 2). Overexpression of miR-1273g-3p increases Aβ production; Figure S2 (related to Figure 3). Expressions of APP and nicastrin are not regulated by oxidative stress in miR-1273g-3p overexpressing H4-APPswe cells; Figure S3 (related to Figure 4). Overexpression of miR-1273g-3p impairs mitochondrial function; Figure S4 (related to Figure 5). miR-1273g-3p negatively regulates mitochondrial genes; Table S1. Raw data of microarray; Table S2. Ct values of qPCR analysis of plasma miRNAs; Table S3. Ct values of qPCR analysis of CSF miRNAs; Table S4. Sequences of PCR primers.
