# Supplementary material for: Enhanced Expression of microRNA-1273g-3p Contributes to Alzheimer’s Disease Pathogenesis by Regulating the Expression of Mitochondrial Genes
Source: Cells. 2021 Oct 9;10(10):2697. doi: 10.3390/cells10102697 (PMC8534383; doi:10.3390/cells10102697)
Supplement: Supplementary file 1 [file cells-10-02697-s001.zip › Supplementary figures-revised-final.pdf]

Supplementary Figure S1

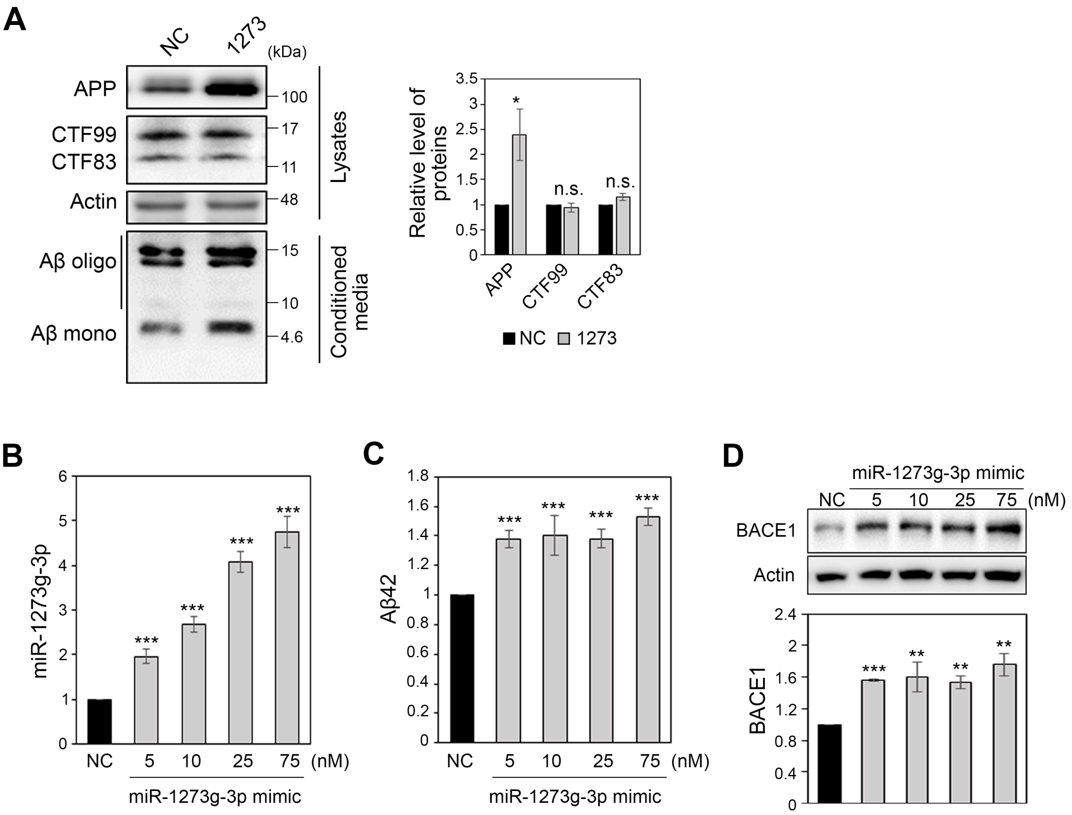

Figure S1. | (related to Figure 2). Overexpression of miR-1273g-3p increases Aβ production. (**A**) Western blotting assays of the levels of amyloid precursor protein (APP) and c-terminal fragments (CTFs) in lysates and Aβ in conditioned medium of H4-APPswe cells transfected with miR-1273g-3p mimic or negative control. Bar graphs show densitometric results for APP and CTFs. Data were normalized relative to expression of actin (n = 3). (**B**) The level of miR-1273g-3p was measured by qPCR in H4-APPswe cells transfected with 5, 10, 25 and 75 nM miR-1273g-3p mimic. Data were normalized relative to RNU6 (n = 3 each). **(C)** Enzyme-linked immunosorbent assay (ELISA) of Aβ42 and Aβ40 in conditioned media of H4-APPswe cells transfected with 5, 10, 25 and 75 nM miR-1273g-3p mimic or negative control (n = 3 each). (**D**) Western blotting for BACE1 in H4-APPswe cells transfected with miR-1273g-3p mimic or negative control. Bar graphs show densitometric results for BACE1. Data were normalized relative to actin (n = 3 each). All data are presented as mean ± SEM. ** $p < 0.01$; *** $p < 0.001$; n.s., non-significant (Student's *t*-test). NC, mimic negative control.

Supplementary Figure S2

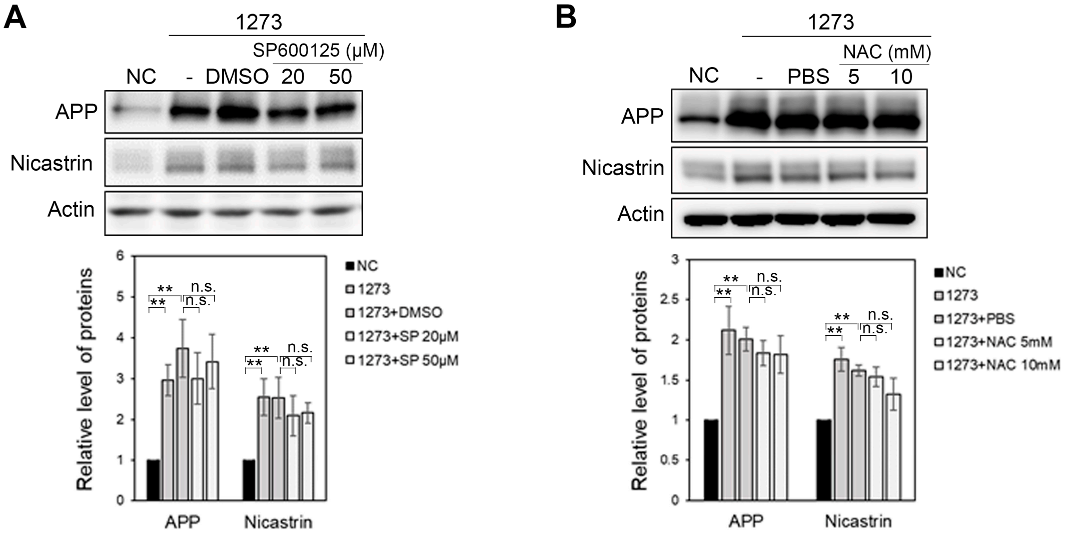

Figure S2. | (related to Figure 3). Expressions of APP and nicastrin are not regulated by oxidative stress in miR-1273g-3p overexpressing H4-APPswe cells. (**A**) SP600125 inhibition of JNK activation in miR-1273g-3p-overexpressing H4-APPswe cells. Cell lysates were analyzed by Western blotting against APP and nicastrin. Bar graphs show densitometric results for APP and CTFs. Data were normalized relative to the expression of actin (n = 3). (**B**) Effects of the antioxidant N-acetylcysteine (NAC) on the expressions of APP and nicastrin by H4-APPswe cells overexpressing miR-1273g-3p, as determined by Western blotting. Bar graphs show densitometric results for APP and CTFs. Data were normalized relative to the expression of actin (n = 3). All data are presented as mean ± SEM. * $p < 0.05$, ** $p < 0.01$, ***$p < 0.001$, n.s., non-significant (Student's *t*-test). NC, mimic negative control; 1273, miR-1273g-3p mimic; SP, SP600125.

Supplementary Figure S3

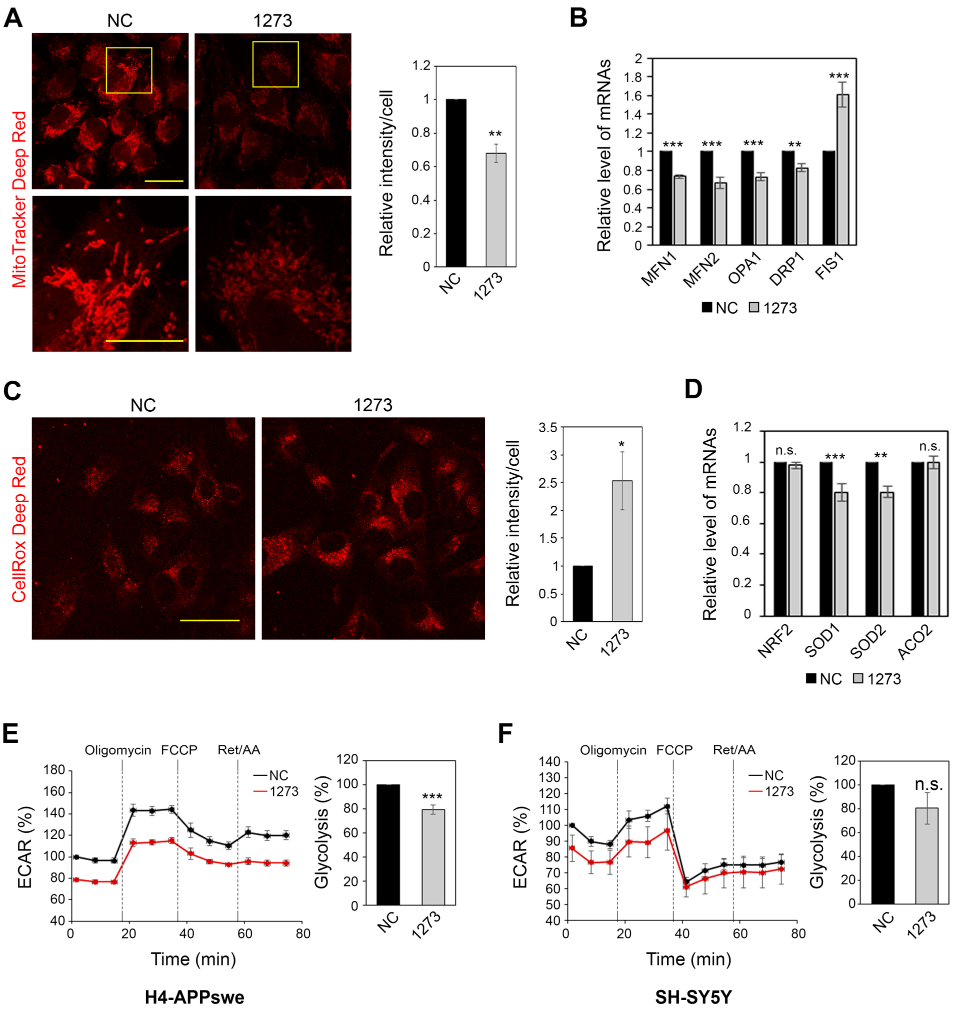

Figure S3. | (related to Figure 4). Overexpression of miR-1273g-3p impairs mitochondrial function. (**A**) Representative images of MitoTracker™ Deep Red FM stained H4-APPswe cells transfected with miR-1273g-3p mimic or negative control. Magnified views of the boxed area are presented in the second row. Scale bar: first row, 20 µm; second row, 10 µm. The bar graph shows the intensity of MitoTracker™ Deep Red FM staining (n = 3, 39-63 cells analyzed per experiment). (**B**) qPCR quantification of the mRNA levels of MFN1, MFN2, OPA1, DRP1 and FIS1 in H4-APPswe cells transfected with miR-1273g-3p mimic or negative control. Data were normalized relative to the expression of GAPDH mRNA (n = 5). (C) Representative CellROX™ Deep Red stained images of H4-APPswe cells transfected with miR-1273g-3p mimic or negative control. The bar graphs show the intensity of CellROX™ Deep Red (n = 3, with 60-73 cells analyzed per experiment). (**D**) Quantitative RT-PCR analysis of the levels of NRF2, SOD1, SOD2 and ACO2 mRNAs in H4-APPswe cells transfected with miR-1273g-3p mimic or negative control. Data were normalized relative to the expression of GAPDH mRNA (n = 5). (**E**,**F**) Measurements of extracellular acidification rate (ECAR) while measuring OCR of H4-APPswe (**E**) and SH-SY5Y (**F**) cells transfected with miR-1273g-3p mimic or negative control. The bar graph shows the glycolysis rates (n = 3). All data are presented as mean ± SEM. * $p < 0.05$, ** $p < 0.01$, *** $p < 0.001$, n.s., non-significant (Student's *t*-test). NC, mimic negative control; 1273, miR-1273g-3p mimic.

Supplementary Figure S4

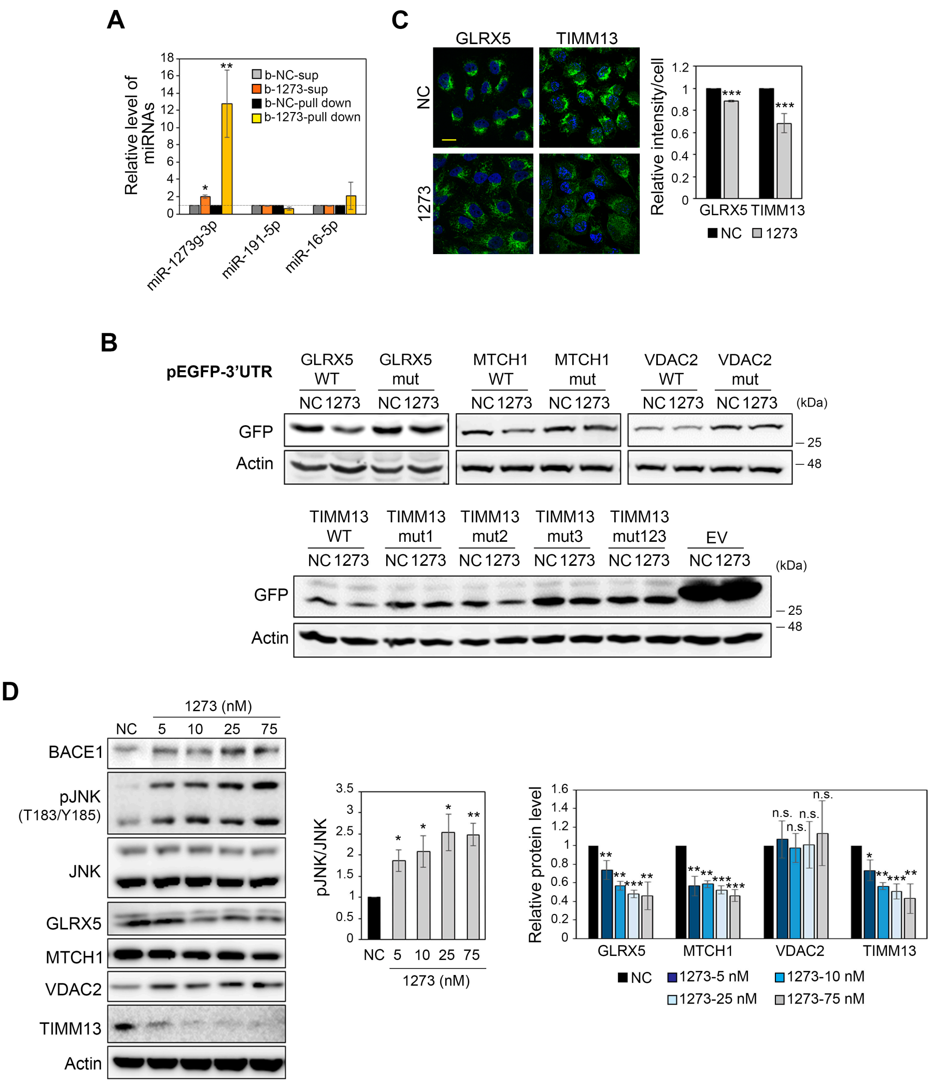

Figure S4. | (related to Figure 5). miR-1273g-3p negatively regulates mitochondrial genes. (**A**) Validation of the specificity and efficiency of pull-down assay performed using biotinylated-miR-1273g-3p and negative control (miR-cel-39-3p) with streptavidin beads, as assessed by qPCR analysis of miR-1273g-3p and the reference miRNAs, miR-191-5p and miR-16-5p. (**B**) GFP expression in H4-APPswe cells co-transfected with miR-1273g-3p mimic or negative control and reporter vectors or empty vector was analyzed by Western blotting after reporter gene assay. (**C**) Immunofluorescence for GLRX5 and TIMM13 in H4-APPswe cells transfected with miR-1273g-3p mimic or negative control. Bars indicate the relative intensity of GLRX5 and TIMM13. (**D**) Western blotting for BACE1, p-JNKT183/Y185, JNK, GLRX5, MTCH1, VDAC2 and TIMM13 in H4-APPswe cells transfected with 5 to 75 nM miR-1273g-3p mimic or negative control. Bar graphs show densitometric results of p-JNK$^{T183/Y185}$ to JNK and GLRX5, MCTH1, VDAC2 and TIMM13. Data were normalized relative to actin (n = 3 each). All data are presented as mean ± SEM. * $p < 0.05$, ** $p < 0.01$, *** $p < 0.001$ (Student's *t*-test). b-NC-sup, supernatant of pull-down sample with biotinylated negative control; b-1273-sup, supernatant of pull-down sample with biotinylated miR-1273g-3p; b-NC-pull-down, pull-down sample of biotinylated negative control; b-1273-pull-down, pull-down sample of biotinylated miR-1273g-3p; NC, mimic negative control; 1273, miR-1273g-3p mimic.
