# Supplementary material for: Enhanced Expression of microRNA-1273g-3p Contributes to Alzheimer’s Disease Pathogenesis by Regulating the Expression of Mitochondrial Genes
Source: Cells. 2021 Oct 9;10(10):2697. doi: 10.3390/cells10102697 (PMC8534383; doi:10.3390/cells10102697)
Supplement: Supplementary file 1 [file cells-10-02697-s001.zip › Supplementary Methods-revised.pdf]

1. Supplementary Materials and Methods

1-1. Enzyme linked immunosorbent assay (ELISA)

H4-APPswe cells were transfected with miRNA mimics or siRNAs and the media was changed with new culture media without antibiotics after 24 h. Conditioned medium was harvested after incubation for 24 h. Aβ peptides in conditioned medium were quantified using Aβ40 or Aβ42 ELISA kit (Invitrogen, USA) according to manufacturer's instruction. Concentration of Aβ peptides was normalized by total protein concentration.

1-2. Western blotting

The cells were incubated for 48hr after transfection and lysed with radioimmunoprecipitation assay buffer containing 50 mM Tris-HCl pH 7.4, 150 mM NaCl, 1% NP40, 0.5% sodium deoxycholate, 0.1% sodium dodecyl sulfate, 10 mM NaF, 1 mM Na_3VO_4 and protease inhibitor cocktail (Roche). The antibodies against APP c-term (Sigma-Aldrich, USA), nicastrin (Cell signaling, USA), ADAM10 (Santa Cruz Biotechnology, USA), Aβ (6E10, covance, USA), BACE1 (Sigma-Aldrich), PS1 (Millipore, USA), p-Tau^{T231} (Cell Signaling), p-Tau^{S396}(Cell Signaling), Tau (Life technologies, USA), p-JNK $^{T183/Y185}$ (Cell Signaling), JNK (Cell Signaling), GLRX5 (Abcam, UK), MTCH1 (Abcam), VDAC2 (Invitrogen), TIMM13 (Novus, USA), GFP (generated in our lab) and β-Actin (Santa Cruz Biotechnology) were used. Densitometric analyses were performed using Image J software (National Institutes of Health, USA) and normalized by actin.

1-3. Reporter gene assay

GFP Reporter vectors of 3'UTR of GLRX5, MTCH1, VDAC2 and TIMM13 were transfected into H4-APPswe cells, and after 6 h, miR-1273g-3p mimic or negative control was transfected into the cells. After that, the cells were further incubated for 48 h followed by western blot analysis for GFP.

1-4. Measurements of H_2O_2

Concentration of intracellular H_2O_2 was measured using Fluorimetric Hydrogen Peroxide Assay Kit (Sigma-Aldrich) according to manufacturer's protocol. Fluorescence intensity at (λ_{ex}=540/ λ_{em}=590 nm)

were analyzed using SpectraMax M2 (Molecular devices, USA). Data were normalized by total protein concentration.

1-5. Active mitochondria staining

H4-APPswe or SH-SY5Y cells cultured on the 18 mm cover glasses were transfected with miRNA mimics and incubated for 48hr. Then, the cells were incubated with culture media containing 200 nM of MitoTracker Red CMXRos (Invitrogen, USA) for 20 min in a humidified incubator containing 95% air/5% CO_2 at 37 °C and fixed using 4% paraformaldehyde for 15 min at 37 °C.

1-6. Immunofluorescence

H4-APPswe cells cultured on 18 mm cover glasses were transfected with miRNA mimics and incubated for 48 h. After that, the cells were fixed with 4% PFA, permeabilized with 0.1% TritonX-100 and incubated with primary antibodies of GLRX5 (Abcam), TIMM13 (Novus) or MAP2 after blocking with 1% BSA, followed by Alexa 488-conjugated secondary antibody (Molecular Probes, USA).
